# Impact of Ultra-Rapid Insulin on Boost and Ease-Off in the Cambridge Hybrid Closed-Loop System for Individuals With Type 1 Diabetes

**DOI:** 10.1177/19322968241289963

**Published:** 2024-10-18

**Authors:** Chloë Royston, Charlotte Boughton, Munachiso Nwokolo, Rama Lakshman, Sara Hartnell, Malgorzata E. Wilinska, Julia Ware, Janet M. Allen, Hood Thabit, Julia K. Mader, Lia Bally, Lalantha Leelarathna, Mark L. Evans, Roman Hovorka

**Affiliations:** 1 Metabolic Research Laboratories, Wellcome-MRC Institute of Metabolic Science, University of Cambridge, Cambridge, UK; 2 Wolfson Diabetes and Endocrine Clinic, Cambridge University Hospitals NHS Foundation Trust, Cambridge, UK; 3 Royal Free London NHS Foundation Trust, London, UK; 4 Department of Paediatrics, University of Cambridge, Cambridge, UK; 5 Diabetes, Endocrinology and Metabolism Centre, Manchester Royal Infirmary, Manchester University NHS Foundation Trust, Manchester, UK; 6 Division of Endocrinology and Diabetology, Department of Internal Medicine, Medical University of Graz, Graz, Austria; 7 Department of Diabetes, Endocrinology, Nutritional Medicine and Metabolism, Inselspital, Bern University Hospital, Bern, Switzerland; 8 Department of Metabolism, Digestion and Reproduction, Imperial College London, London, UK

**Keywords:** boost, ease-off, hybrid closed-loop, rapid-acting insulin, type 1 diabetes, ultra–rapid-acting insulin

## Abstract

**Objective:**

The objective was to evaluate the safety and efficacy of ultra–rapid-acting insulin with the Boost and Ease-off features of the Cambridge hybrid closed-loop system.

**Methods:**

A secondary analysis of Boost and Ease-off from two double-blind, randomized, crossover hybrid closed-loop studies comparing (1) Fiasp to insulin aspart (n = 25), and (2) Lyumjev to insulin lispro (n = 26) was carried out. Mean glucose on initialization of Boost and Ease-off, change in glucose 60 and 120 minutes after initialization, duration and frequency of use, mean glucose, and time in, above, and below target glucose range were calculated for periods of Boost use, Ease-off use, or neither.

**Results:**

Participants used Boost for longer with Fiasp than insulin aspart (median [interquartile range, IQR] = 75 [53-125] minutes vs 60 [49-75] minutes; *P* = .01). Mean glucose on Boost initialization with Fiasp was 238 ± 62 mg/dL compared with 218 ± 45 mg/dL with insulin aspart (*P* = .08). Fiasp use resulted in a greater glucose reduction 120 minutes after Boost initialization [−59 ± 34 mg/dL vs −43 ± 31 mg/dL; *P* = .02]. There were no statistically significant differences in sensor glucose endpoints during Boost or Ease-off periods between Fiasp and aspart. There were no statistically significant differences during Boost or Ease-off periods when comparing Lyumjev with insulin lispro. There were no safety issues when using Boost and Ease-off with ultra-rapid insulins.

**Conclusions:**

The use of Fiasp and Lyumjev during Boost or Ease-off resulted in comparable safety and efficacy to using insulin aspart and lispro.

## Introduction

Hybrid closed-loop insulin delivery systems have become the standard of care to manage type 1 diabetes.^1^ These systems apply glucose-responsive insulin delivery improving glycemic outcomes including increasing time in range without increasing the risk of hypoglycemia, and improving quality of life.^2^ A limiting factor of automated insulin delivery systems is the relatively slow absorption of rapid-acting insulin analogs, which leads to less effective glucose lowering and greater glycemic fluctuations.^3^

Ultra-rapid insulins such as Fiasp and Lyumjev have been developed to speed up absorption. Fiasp (a niacinamide and L-arginine containing insulin aspart) has an onset that is 5 minutes faster than standard insulin aspart, whereas Lyumjev (a treprostinil and citrate containing insulin lispro) has an onset that is 11 minutes faster than standard insulin lispro in individuals with type 1 diabetes.^4^ Although some closed-loop studies have shown small improvements in glucose outcomes with Fiasp^5^ and Lyumjev,^6^ outcomes have been inconsistent.^7-10^

The Cambridge hybrid closed-loop system comprising the interoperable CamAPS FX app, a compatible continuous glucose monitor (CGM) and a compatible insulin pump, has two features, Boost and Ease-off. These features provide enhanced customizability to the users at times of higher or lower insulin needs, by using altered insulin sensitivity and/or glucose targets to adjust how much insulin is delivered. These features can be initiated by the user to reduce the risk of hypoglycemia during periods of increased insulin sensitivity such as exercise (Ease-off) and to improve glucose outcomes when insulin sensitivity is lower such as during intercurrent illness, stress, or hormonal changes associated with the menstrual cycle or times of unusual hyperglycemia (Boost).^11^ There are currently no studies investigating the use of these features with ultra–rapid-acting insulins. The objective of this study was to analyze whether the use of ultra–rapid-acting insulins (Fiasp and Lyumjev) improves the safety and efficacy of Boost and Ease-off (compared with standard insulin aspart and insulin lispro, respectively).

## Materials and Methods

### Study Design and Population

A secondary analysis of two double-blind, randomized, crossover hybrid closed-loop studies that compared the safety and efficacy of Fiasp to standard insulin aspart (Novo Nordisk, Bagsvaerd, Denmark) and Lyumjev to standard insulin lispro (Eli Lilly, Indianapolis, Indiana) was carried out. Each participant spent two 8-week periods on the hybrid closed-loop system using Fiasp and insulin aspart^7^ or Lyumjev and insulin lispro.^6^ Inclusion criteria for both studies included a diagnosis of type 1 diabetes as defined by the World Health Organization, an age of ≥ 18 years old, an HbA_1c_ of ≤ 10% (86 mmol/mol), and the use of an insulin pump for at least 6 months. Exclusion criteria included more than one episode of severe hypoglycemia in the previous 12 months, a total daily dose (TDD) of ≥ 2.0units/kg/day and pregnancy. Independent research ethics committees approved both studies. Participants signed informed consent before any study-related activity.

### Hybrid Closed-Loop System

Participants in both studies used the Cambridge interoperable hybrid closed-loop app with the Dexcom G6 (Dexcom, San Diego, California) CGM and DANA Diabecare RS or DANA-I insulin pumps (Sooil, Seoul, Republic of Korea). The app, installed on a standard compatible Android smartphone, uses an adaptive model predictive control algorithm for titrating insulin delivery.^12^ The algorithm is initialized by entering user’s weight and TDD. Insulin sensitivity and active insulin time are automatically calculated and adjusted as necessary. The advanced control algorithm is highly adaptive adjusting total daily insulin requirements, diurnal variations, and insulin delivery around meals. The default target is 104 mg/dL, but this can be adjusted between 80 and 198 mg/dL in segments of 30 minutes.^13^

The hybrid closed-loop app has two features, Boost and Ease-off, providing enhanced customizability to the users at times or higher or lower insulin needs. During Boost use, the control algorithm increases the amount of insulin delivered by assuming higher insulin needs corresponding to approximately a 35% increase of the TDD in a glucose-responsive manner, whereas during Ease-off, the glucose target and insulin sensitivity are increased causing the control algorithm to deliver less insulin.

Users are advised that Boost can be used when insulin requirements are higher (eg, during illness, growth, certain times of the menstrual cycle or times of unusual hyperglycemia), while Ease-off can be used during periods when less insulin is needed and/or insulin sensitivity is higher (eg, during exercise or times of increased hypoglycemia risk).^11^

### Data Analysis

Each sensor glucose reading was determined to be either in Boost mode, Ease-off mode, or neither mode. Only data from periods where closed-loop mode was turned on were included. The change in glucose endpoints was calculated per episode.

Key endpoints included mean sensor glucose at initialization of Boost or Ease-off and the change in sensor glucose at 60 and 120 minutes after initialization for both features. Mean duration and frequency of use of both features was determined. Mean sensor glucose, time spent in range (70-180 mg/dL), time below 70 mg/dL, below 54 mg/dL, above 180 mg/dL, and above 250 mg/dL were calculated for periods with Boost, Ease-off, and neither feature for all four insulin types (Fiasp, insulin aspart, Lyumjev, and insulin lispro).

Endpoints were calculated per participant for each period. Endpoints were compared using paired *t*-test; non-normally distributed endpoints were winsorized at the 10th and 90th percentile prior to statistical inference. To control for multiple comparisons, *P* values were adjusted using the Benjamini-Hochberg method; all adjusted values less than .05 were considered significant.

Data analysis was completed using R Studio (R Foundation for Statistical Computing, Vienna, Austria). Data are presented as mean ± standard deviation for normally distributed data or median (interquartile range) otherwise.

## Results

### Insulin Aspart vs Fiasp

**Figure 1.**
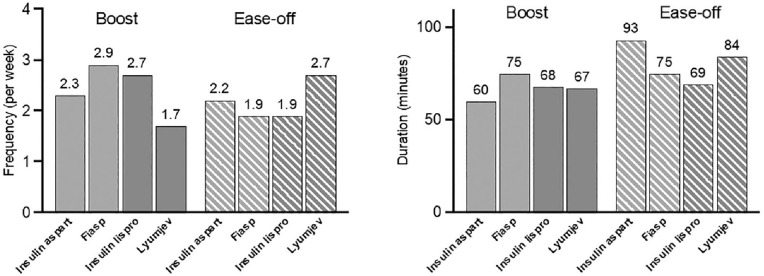
Left: frequency of Boost and Ease-off. Right: duration of Boost and Ease-off.

A total of 25 participants (mean ± SD age 38 ± 9 years, 52% female with diabetes duration 22 ± 12 years and baseline HbA_1c_ 7.4 ± 0.8% [57 ± 8 mmol/mol]) were included in the study. Participants used Boost for longer periods with Fiasp (median [interquartile range, IQR] = 75 [53-125] minutes compared with insulin aspart 60 [49-75] minutes; *P* = .01). Frequency of Boost use with Fiasp was 2.9 (0.5-5.0) times per week, whereas Boost use with insulin aspart was 2.3 (1.8-5.9) times per week (*P* = .66) (Figure 1). Mean glucose on Boost initialization was 238 ± 62 mg/dL with Fiasp compared with 218 ± 45 mg/dL with insulin aspart (*P* = .08). There was no difference in the change in glucose 60 minutes post initialization of Boost between Fiasp and insulin aspart (−16 mg/dL [−41, −4] vs −14 mg/dL [−30, 5]; *P* = .32), but there was a significantly greater reduction in glucose at 120 minutes with Fiasp than with insulin aspart (−60 ± 34 mg/dL vs −43 ± 31 mg/dL; *P* = .02). There were no significant differences between Fiasp and insulin aspart in time in target glucose range, time above 180 mg/dL, or below 70 mg/dL during Boost periods (Table 1).

**Table 1.** Sensor Glucose Outcomes During Boost On and Off Periods With Rapid-Acting Insulin vs Ultra–Rapid-Acting Insulin.

	Insulin aspart	Fiasp	*P*	Insulin lispro	Lyumjev	*P*
Number of participants	25	25	-	26	26	-
Number of analyzed days per user	55 (1)	55 (0)	-	55 (0)	55 (0)	-
CGM and closed-loop usage						
Time using CGM (%)	96 ^(5)^	96 ^(7)^	-	98 ^(1)^	97 ^(5)^	-
Time in closed-loop (%)	96 (94 to 97)	95 (94 to 97)	-	97 (95 to 98)	97 (96 to 97)	-
Boost usage						
Mean duration of Boost (min)	60 (49 to 75)	75 (53 to 125)	.01	68 (58 to 87)	67 (58 to 92)	.94
Frequency of Boost use (per week)	2.3 (1.8 to 5.9)	2.9 (0.5 to 5.0)	.66	2.7 (1.4 to 4.2)	1.7 (0.8 to 4.1)	.37
Mean glucose on initialization (mg/dL)	218 ^(45)^	238 ^(62)^	.08	243 ^(50)^	232 ^(50)^	.48
Change in glucose 60 minutes after initialization (mg/dL)	−14 (−30 to 5)	−16 (−41 to −4)	.32	−27 (−34 to −14)	−22 (−38 to −7)	.56
Change in glucose 120 minutes after initialization (mg/dL)	−43 ^(31)^	−59 ^(34)^	.02	−66 ^(39)^	−58 ^(40)^	.68
Sensor glucose: Boost on						
Mean glucose (mg/dL)	213 ^(37)^	223 ^(56)^	.64	228 ^(45)^	221 ^(42)^	.81
Time in range 70-180 mg/dL (%)	32 ^(25)^	31 ^(25)^	.64	25 ^(26)^	29 ^(29)^	.75
Time <70 mg/dL (%)	0.0 (0.0 to 0.0)	0.0 (0.0 to 0.4)	.13	0.0 (0.0 to 0.3)	0.0 (0.0 to 0.0)	.05
Time <54 mg/dL (%)	0.0 (0.0 to 0.0)	0.0 (0.0 to 0.0)	.45	0.0 (0.0 to 0.0)	0.0 (0.0 to 0.0)	.29
Time >180 mg/dL (%)	68 ^(25)^	69 ^(25)^	.66	74 ^(27)^	71 ^(29)^	.75
Time >250 mg/dL (%)	21 (8 to 39)	23 (16.6 to 39.2)	.12	37 (11 to 53)	30 (15 to 51)	.89
Sensor glucose: Boost off						
Mean glucose (mg/dL)	143 ^(14)^	144 ^(14)^	.33	144 ^(16)^	141 ^(15)^	.24
Time in range 70-180 mg/dL (%)	76 ^(8)^	76 ^(8)^	.57	77 ^(10)^	80 ^(10)^	.03
Time <70 mg/dL (%)	2.8 (1.6 to 4.3)	2.4 (1.1 to 3.1)	.15	2.0 (1.3 to 3.1)	2.2 (1.3 to 2.6)	.41
Time <54 mg/dL (%)	0.5 (0.1 to 0.9)	0.3 (0.2 to 0.7)	.19	0.3 (0.1 to 0.4)	0.3 (0.2 to 0.5)	1.00
Time >180 mg/dL (%)	20 ^(8)^	21 ^(9)^	.22	20 ^(10)^	18 ^(10)^	.09
Time >250 mg/dL (%)	4.4 (2.7 to 6.0)	4.5 (2.0 to 6.4)	.85	3.0 (1.3 to 7.6)	2.9 (1.4 to 5.8)	.61

Data are presented as mean (standard deviation) or median (IQR).

**Figure 2.**
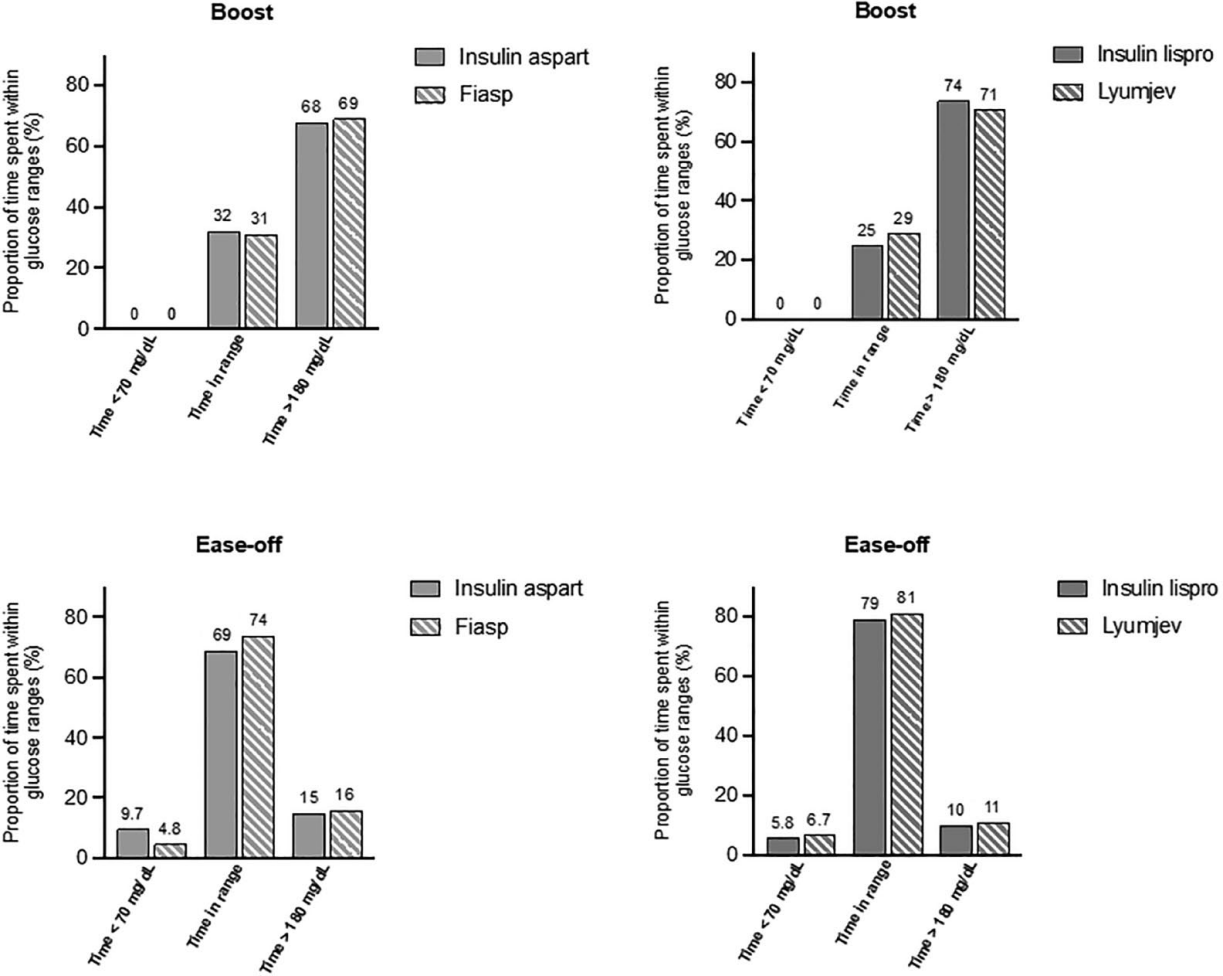
Time above, below, and within target range (between 70 and 180 mg/dL) for Boost with insulin aspart vs Fiasp (top left), for Boost with insulin lispro vs Lyumjev (top right), for Ease-off with insulin aspart vs Fiasp (bottom left), and for Ease-off with insulin lispro vs Lyumjev (bottom right).

There was no difference between Fiasp and insulin aspart in the frequency or duration of Ease-off usage (Figure 1), mean glucose on Ease-off initialization, or change in glucose 60 and 120 minutes after initialization. During Ease-off periods, there were no statistically significant differences between Fiasp and insulin aspart in CGM endpoints (Figure 2). There were no events of severe hypoglycemia or diabetic ketoacidosis.

### Insulin Lispro and Lyumjev

There were 26 participants (mean ± SD age 44 ± 11 years, 36% female with diabetes duration 30 ± 12 years and baseline HbA_1c_ 7.1 ± 0.9% [54 ± 10 mmol/mol]) included in the study. There were no statistically significant differences when comparing Lyumjev with insulin lispro during Boost or Ease-off periods (Tables 1 and 2). Boost was used 1.7 (0.8-4.1) times per week with Lyumjev compared with 2.7 (1.4-4.2) times per week with insulin lispro (*P* = .37), and duration of Boost usage was similar for Lyumjev compared with insulin lispro (67 [58-92] minutes compared with 68 [58-87] minutes; *P* = .94) (Figure 1). Frequency and duration of use for Ease-off with Lyumjev were 2.7 (0.8-4.3) times per week and 84 (65-90) minutes, respectively, compared with 1.9 (0.6-4.7) times per week (*P* = .78) and 69 (54-104) minutes (*P* = .55) with insulin lispro (Figure 2).

**Table 2.** Sensor Glucose Outcomes During Ease-Off On and Off Periods With Rapid-Acting Insulin vs Ultra–Rapid-Acting Insulin.

	Insulin aspart	Fiasp	*P*	Insulin lispro	Lyumjev	*P*
Number of participants	25	25	-	26	26	-
Number of analyzed days per participant	55 ^(1)^	55 (0)	-	55 (0)	55 (0)	-
CGM and closed-loop usage						
Time using CGM (%)	96 ^(5)^	96 ^(7)^	-	98 ^(1)^	97 ^(5)^	-
Time in closed-loop (%)	96 (94 to 97)	95 (94 to 97)	-	97 (95 to 98)	97 (96 to 97)	-
Ease-off usage						
Mean duration of Ease-off (min)	93 (56 to 104)	75 (59 to 95)	.35	69 (54 to 104)	84 (65 to 90)	.55
Frequency of Ease-off use (per week)	2.2 (0.9 to 4.7)	1.9 (1.2 to 5.6)	.45	1.9 (0.6 to 4.7)	2.7 (0.8 to 4.3)	.78
Mean glucose on initialization (mg/dL)	109 ^(34)^	113 ^(21)^	.85	106 ^(21)^	109 ^(18)^	.53
Change in glucose 60 minutes after initialization (mg/dL)	11.7 (−5.9 to 31.5)	8.3 (−8.2 to 30.4)	.75	17.5 (7.7 to 29.1)	18.5 (4.6 to 27.8)	.48
Change in glucose 120 minutes after initialization (mg/dL)	40 ^(47)^	42 ^(43)^	.90	47 ^(35)^	48 ^(47)^	.38
Sensor glucose: Ease-off on						
Mean glucose (mg/dL)	125 ^(34)^	127 ^(25)^	.89	116 ^(23)^	122 ^(21)^	.47
Time in range 70-180 mg/dL (%)	69 ^(22)^	74 ^(18)^	.75	79 ^(14)^	81 ^(10)^	.86
Time <70 mg/dL (%)	9.7 (4.2 to 19.6)	4.8 (2.8 to 14.3)	.65	5.8 (2.5 to 13.3)	6.7 (2.4 to 9.8)	.74
Time <54 mg/dL (%)	2.1 (0.3 to 4.0)	1.0 (0.0 to 3.2)	.75	0.9 (0.0 to 2.2)	0.2 (0.0 to 1.1)	.41
Time >180 mg/dL (%)	15 ^(19)^	16 ^(18)^	.88	10 ^(10)^	11 ^(11)^	.99
Time >250 mg/dL (%)	0.0 (0.0 to 1.6)	0.0 (0.0 to 1.2)	.90	0.0 (0.0 to 2.3)	0.0 (0.0 to 2.1)	.63
Sensor glucose: Ease-off						
Mean glucose (mg/dL)	143 ^(14)^	144 ^(14)^	.33	144 ^(16)^	141 ^(15)^	.24
Time in range 70-180 mg/dL (%)	76 ^(8)^	76 ^(8)^	.57	77 ^(10)^	80 ^(10)^	.03
Time <70 mg/dL (%)	2.8 (1.6 to 4.3)	2.4 (1.1 to 3.1)	.15	2.0 (1.3 to 3.1)	2.2 (1.3 to 2.6)	.41
Time <54 mg/dL (%)	0.5 (0.1 to 0.9)	0.3 (0.2 to 0.7)	.19	0.3 (0.1 to 0.4)	0.3 (0.2 to 0.5)	1.00
Time >180 mg/dL (%)	20 ^(8)^	21 ^(9)^	.22	20 ^(10)^	18 ^(10)^	.09
Time >250 mg/dL (%)	4.4 (2.7 to 6.0)	4.5 (2.0 to 6.4)	.85	3.0 (1.3 to 7.6)	2.9 (1.4 to 5.8)	.61

Data are presented as mean (standard deviation) or median (IQR).

Mean glucose on initialization of Boost was 232 ± 50 mg/dL with Lyumjev compared with 243 ± 50 mg/dL with insulin lispro (*P* = .48) and change in glucose after 60 or 120 minutes was similar for Lyumjev and insulin lispro (18.5 mg/dL [4.6-27.8] with Lyumjev vs 17.5 mg/dL [7.7-29.1] with insulin lispro; *P* = .48 after 60 minutes and 48 ± 47 mg/dL with Lyumjev vs 47 ± 35 mg/dL with insulin lispro; *P* = .38 after 120 minutes).

Mean glucose on initialization of Ease-off, change in glucose after 60 and 120 minutes, and sensor glucose endpoints were similar for both insulin types (Table 2). There were no events of severe hypoglycemia or diabetic ketoacidosis.

## Discussion

Our analysis suggests that ultra-rapid insulins Fiasp and Lyumjev show similar algorithm responsiveness during Boost or Ease-off periods compared with standard rapid-acting insulins. There were no statistically significant differences in sensor glucose endpoints during Boost or Ease-off periods when comparing Fiasp and Lyumjev to insulin aspart and lispro, respectively.

During Boost and Ease-off periods, all four insulins produced a relatively small change in glucose over the 60 minutes, but the magnitude of change increased (between 2 and 5 fold greater) at 120 minutes, which suggests it may take longer than 60 minutes to see a significant change in glucose levels with Boost and Ease-off, irrespective of whether fast-acting or ultra-rapid insulin is used. Studies show that the peak effect of insulin is reached between 90 and 180 minutes, depending on the insulin type and individual physiology.^4^

We observed that Boost was started at a higher glucose with Fiasp compared with insulin aspart and used for a longer duration, which is likely to account for the increased change in glucose at 120 minutes after initialization with Fiasp compared with insulin aspart. There is some research suggesting that unexplained hyperglycemia is more common in insulin pump users using Fiasp compared with insulin aspart,^14^ which may explain the observed differences in Boost usage.

There was a tendency toward a higher frequency and longer duration of use for Ease-off with Lyumjev, suggesting that users may have felt the need to prevent hypoglycemia more often when using Lyumjev compared with insulin lispro. As both studies were blinded, participant’s decisions could not be biased based on knowledge of which insulin was being used.

Importantly, there do not appear to be any safety issues when using Boost and Ease-off with ultra-rapid insulins. There was no hypoglycemia during Boost periods with either Fiasp or insulin aspart, and no increased risk of hyperglycemia during Ease-off with Fiasp. The same applies for Lyumjev and insulin lispro. This observation, combined with the fact that there were no severe hypoglycemic or diabetic ketoacidosis events, provides reassuring data for the use of Boost and Ease-off with ultra-rapid insulins.

Strengths of the present analyses include the double-blinded crossover study design. Participants served as their own controls, and there could be no bias on decisions on whether to use Boost or Ease-off. Limitations include the exploratory nature of this study affecting interpretation of the results, the small sample size, participants with low baseline HbA_1c_ (7.4% for the aspart study and 7.1% for lispro) and a lack of ethnic diversity in the study population, which limits generalizability. A further limitation is the lack of data collection on participants’ behavioral patterns such as food intake or physical activity. These patterns may have been different in the different insulin arms, which could impact the usage or efficacy of Boost and Ease-off.

## Conclusion

In conclusion, the use of Fiasp and Lyumjev during Boost or Ease-off resulted in comparable efficacy of hybrid closed-loop insulin delivery to using insulin aspart and lispro. The use of ultra-rapid insulins in the Cambridge hybrid closed-loop system did not increase the risk of hypoglycemia during Boost periods or the risk of hyperglycemia during Ease-off periods.
